# Structure-based functional annotation of hypothetical proteins from *Candida dubliniensis:* a quest for potential drug targets

**DOI:** 10.1007/s13205-014-0256-3

**Published:** 2014-10-17

**Authors:** Kundan Kumar, Amresh Prakash, Farah Anjum, Asimul Islam, Faizan Ahmad, Md. Imtaiyaz Hassan

**Affiliations:** 1 Center for Interdisciplinary Research in Basic Sciences, Jamia Millia Islamia, Jamia Nagar, New Delhi, 110025 India; 2 Female College of Applied Medical Science, Taif University, Al-Taif, Kingdom of Saudi Arabia

**Keywords:** *Candida dubliniensis*, Hypothetical protein, Sequence analysis, Homology modeling, Functional annotation, Domains and motifs, Functional genomics

## Abstract

*Candida dubliniensis* is an emerging pathogenic yeast in humans and infections are usually restricted to mucosal parts of the body. However, its presence in specimens of immunocompromised individuals, especially in HIV-positive patients, is of major medical concern. There is a large fraction of genomes of *C. dubliniensis* in the database which are uncharacterized for their biochemical, biophysical, and/or cellular functions, and are identified as hypothetical proteins (HPs). Function annotation of *Candida* genome is, therefore, essentially required to facilitate the understanding of mechanisms of pathogenesis and biochemical pathways important for selecting novel therapeutic target. Here, we carried out an extensive analysis to explain the functional properties of genome, using available protein structure and function analysis tools. We successfully modeled the structures of eight HPs for which a template with moderate sequence similarity was available in the protein data bank. All modeled structures were analyzed and we found that these proteins may act as transporter, kinase, transferase, ketosteroid, isomerase, hydrolase, oxidoreductase, and binding targets for DNA and RNA. Since these unique HPs of *Candida* showed no homologs in humans, these proteins are expected to be a potential target for future antifungal therapy.

**Electronic supplementary material:**

The online version of this article (doi:10.1007/s13205-014-0256-3) contains supplementary material, which is available to authorized users.

## Introduction

In recent years, *Candida dubliniensis* has emerged as a major cause of candidal infection in humans, particularly in HIV-infected patients and other immunocompromised persons (Sullivan and Coleman 1998). Normally, this species of *Candida* is harmless, but it may become infectious under certain circumstances and is thus also called an opportunistic pathogen (Butler et al. 2009). In fact, *C. dubliniensis* is substantially less pathogenic than its closely related species *C. albicans* (Karkowska-Kuleta et al. 2009). However, a longer survival time and adaptability in the host induce virulence (Stokes et al. 2007). Furthermore, the genotypic and phenotypic closeness with *C. albicans*, misguides the medical practitioners, in routine laboratory diagnosis, and its treatment becomes tougher with their tendency to develop resistance against antifungal agents (Moran et al. 1997). In general, *C.* *dubliniensis* infection is restricted to oral mucosa, vagina and lungs. However, it may cause fatal systemic infection (Sullivan et al. 2005). Therefore, this specie is under investigation during fungal infection, especially in the condition of low immunity patient (Achkar and Fries 2010).

The complete genome of *C. dubliniensis* has been sequenced recently, and it consists of eight chromosomal contigs with 262288 reads of total size 14.6 Mb (Jackson et al. 2009). Extensive analysis of 5,860 open reading frames leads to identification of 1,273 hypothetical proteins (HPs), whose functions have not been determined so far (Galperin and Koonin 2004). Function determination of putative uncharacterized HPs for their possible biological activity has emerged as an important focus for computational biology (Kumar et al. 2014; Loewenstein et al. 2009; Shahbaaz et al. 2013). The primary approach is to assign a function to new genes based on sequence homology (Desler et al. 2009). Various HPs are found to be well conserved among organisms and are involved in important biochemical processes (Dutta et al. 2013). Further, the characterization of protein function in context of their sequence similarity is a primitive approach and may lead to ambiguous function annotation (Chiusano et al. 2008). In various cases, the evolution retains a conserved folding pattern despite having very poor sequence similarity (Chiusano et al. 2008). Therefore, the structural analysis of protein is essential to decipher their biochemical functions that could not be illustrated from sequence data alone (Ebihara et al. 2006; Hassan et al. 2008, 2013). Hence, the three-dimensional structure determination of HPs is an imperative task to illustrate the biological function at the molecular level (Shahbaaz et al. 2014; Sinha et al. 2014).

Recently, we annotated functions of several HPs from *Candida dubliniensis* (Kumar et al. 2014). To extend this study further, we modeled the structure of those proteins for which we got sufficient sequence similarity and coverage to further improve the function assignment. We have already been involved in structure-based drug design (Hassan et al. 2007a, b; Prakash et al. 2013; Thakur and Hassan 2011; Thakur et al. 2013) and hence we are looking for novel therapeutic targets (Shahbaaz et al. 2013). To validate a potential drug target, protein–ligand docking studies have been proven as one of the appropriate tools (Singh et al. 2014; Tasleem et al. 2014; Thakur et al. 2014; Totrov and Abagyan 2008). Hence, we docked few drug molecules with HPs as well. A precise annotation of HPs from *C. dubliniensis* may lead to the identification of new functions, and novel pharmacological targets for drug design, discovery and screen to cure the candidal infections.

## Materials and methods

### Sequence retrieval and analysis

*Candida dubliniensis* genome encodes 5,860 proteins, wherein functions are uncharacterized for 1,273 proteins and are termed as HPs (O’Connor et al. 2010). Sequences of HPs from *C. dubliniensis* were retrieved from UniProt database (http://www.uniprot.org/), and BLAST (Altschul et al. 1990) and PSI-BLAST (Altschul et al. 1997) searches were carried out to identify similar sequences with known structures and functions. We extensively analyzed functions using conserved domain database (CDD) (Marchler-Bauer et al. 2011), InterProScan (Quevillon et al. 2005) and superfamily databases (Marchler-Bauer et al. 2011). InterProScan (Quevillon et al. 2005) combines different protein signature recognition methods from the InterPro consortium for motif discovery. In a protein, the motifs are signatures of protein families and can be preferably used to define the protein function, particularly in enzyme where motifs are associated with the catalytic function (Bork and Koonin 1996). We also used SMART (Letunic et al. 2012), ScanProsite (de Castro et al. 2006; Gattiker et al. 2002), CATH (Orengo et al. 1997) and PANTHER (Thomas et al. 2003) to establish the evolutionary relationships and infer the functions of HPs. All bioinformatics tools and databases used for sequence and structure analysis are listed in the Table 1. FungalRV was used to identify adhesins character to describe the virulence factor for *C. dubliniensis* HPs. (Chaudhuri et al. 2011). This tool is based on the support vector machine (SVM) method and trained by a large number of compositional properties that are used to classify human pathogenic fungal adhesins and adhesins-like protein.

**Table 1:** List of bioinformatics tools and databases used for sequence and structure-based function annotation

S. no.	Name of Web server	URL	Uses	References
1. Sequence similarity search tool				
	BLAST: basic local alignment search tool	http://blast.ncbi.nlm.nih.gov/Blast.cgi	To find a similar sequence in the database	Mount (2007)
2. Biophysical and chemical characterization				
	ProtParam	http://web.expasy.org/protparam/	To calculate physical and chemical properties	Wilkins et al. (1999)
3. Sub-cellular localization of the protein				
I.	SOSUI	http://bp.nuap.nagoya-u.ac.jp/sosui/sosui_submit.html	To predict the transmembrane domain	Hirokawa et al. (1998)
II.	TMHMM	http://www.cbs.dtu.dk/services/TMHMM/	Used to predict the transmembrane topology	Krogh et al. (2001), Sonnhammer et al. (1998)
III.	Psort II	http://psort.hgc.jp/form2.html	To predict sub-cellular localization	Nakai and Horton (1999)
IV.	SignalP	http://www.cbs.dtu.dk/services/SignalP/	To predict cleavage site of signal protein	Petersen et al. (2011)
V.	HMMTOP	http://www.enzim.hu/hmmtop/index.php	To predict the transmembrane helix	Tusnady and Simon (2001)
4. Functional analysis tool				
I.	Conserved domain	http://www.ncbi.nlm.nih.gov/Structure/cdd/wrpsb.cgi	To search conserved domain in the sequences	Marchler-Bauer et al. (2011)
II.	InterProScan	http://www.ebi.ac.uk/Tools/pfa/iprscan/	To find the motif in the sequences	Quevillon et al. (2005)
III.	Interpro	http://www.ebi.ac.uk/interpro/	To categorize by predicting domains and important sites	Apweiler et al. (2001)
IV.	SMART	http://smart.embl-heidelberg.de/	To Identify and annotate domains in protein	Letunic et al. (2012)
V.	CATH	http://www.cathdb.info	To use hierarchical domain classification of PDB structures	Orengo et al. (1997)
VI.	Pfam	http://pfam.sanger.ac.uk	To collect protein families, based on multiple sequence alignments and HMM	Finn et al. (2014)
5. Predicting the fold pattern				
I.	PFP-FunDSeqE	http://www.csbio.sjtu.edu.cn/bioinf/PFP-FunDSeqE/	To find the type of protein fold in the protein sequences	Shen and Chou (2009)
II.	HHpred	http://toolkit.tuebingen.mpg.de/hhpred	Used for homology detection	Kalev and Habeck (2011)
III.	Dali server	http://ekhidna.biocenter.helsinki.fi/dali_server/start	For searching similar 3D structure	Holm and Rosenstrom (2010)
6. Virulence prediction				
	FungalRV	fungalrv.igib.res.in/query.php	In adhesin prediction	Chaudhuri et al. (2011)
7. Structure prediction				
I.	MODELLER	http://salilab.org/modeller/	To model three-dimensional structures	Webb and Sali (2014)
II.	SWISS-MODEL	http://swissmodel.expasy.org/	Homology modeling server	Biasini et al. (2014)
III.	Phyre2	www.sbg.bio.ic.ac.uk/phyre2	Ab initio method for structure prediction	Kelley and Sternberg (2009)
9. Structure validation				
	SAVES	http://autodock.scripps.edu/	To validate protein structures	Laskowski et al. (1996)
10. Docking analysis				
	AutoDock	http://autodock.scripps.edu/		Sandeep et al. (2011)

#### Structure prediction

Three-dimensional structures of HPs are determined using MODELLER (Eswar et al. 2006), SWISS-MODEL (Kiefer et al. 2009) and protein homology recognition engine (Phyre) (Kelley and Sternberg 2009). MODELLER uses the spatial restraints to predict structure, and restraints of the quarry sequence are generated using alignment to related structure. SWISS-MODEL predicts the structure of HP by assembly of rigid bodies, in which a model from a small number of rigid bodies is obtained from the aligned protein structures (Schwede et al. 2003). The Phyre server uses a library of known protein structures taken from the Structural Classification of Proteins (SCOP) database and the PDB (Kelley and Sternberg 2009). The top ten scoring alignments were used to construct the three-dimensional structure of each HP. All predicted models from three sources were subjected to energy minimization with CHARMM-22, using Discovery Studio 3.5 (Hirashima and Huang 2008). Side chains of the predicted model were refined by using side chain refinement protocol of the MODELLER of Discovery studio 3.5.

#### Structure validation

The stereochemical quality of the modeled structure for HPs was validated at structural analysis and with verification server (Laskowski et al. 1993) using PROCHECK (Laskowski et al. 1996). PROCHECK validates the stereochemical quality of a protein structure by analyzing the overall structure and residue-by-residue geometry of proteins. ProQ is a neural network-based predictor server used to define the correctness and quality of predicted structures (Wallner and Elofsson 2003). The model showed higher LG score and MaxSub score that were selected for further analysis. A validation report of each model is listed in Table 2.

**Table 2:** Validation report of all predicted models

S. no.	Uniprot ID	Score	Tools used			Structure selected	Template	RMSD with template
			MODELLER	SWISS-MODEL	Phyre			
1	B9WFH2	LG Score	2.027	0.958	2.061	MODELLER	4GBZ	2.419
		MaxSub	0.116	0.065	0.145			
		Procheck (%)	83.60	74.40	84.60			
2	B9WFH4	LG Score	2.253	3.359	3.576	SWISS-MODEL	3T5P	2.389
		MaxSub	0.152	0.285	0.298			
		Procheck (%)	85.00	83.80	79.60			
3	B9WFR9	LG Score	0.618	2.461	3.357	SWISS-MODEL	3UBM	0.178
		MaxSub	0.020	0.216	0.298			
		Procheck (%)	80.80	87.30	79.60			
4	B9WFS0	LG Score	2.341	1.878	2.667	MODELLER	2RGQ	0.460
		MaxSub	0.309	0.255	0.335			
		Procheck	85.80	84.60	85.80			
5	B9WFS1	LG Score	5.560	5.353	5.137	MODELLER	1VA4	0.296
		MaxSub	0.533	0.495	0.446			
		Procheck (%)	86.60	79.60	87.80			
6	B9WFS6	LG Score	0.599	1.935	5.206	Phyre	1K3R	0.000
		MaxSub	0.012	0.128	0.367			
		Procheck (%)	63.10	74.10	85.10			
7	B9WFU3	LG Score	1.123	1.125	1.622	MODELLER	3IDV	1.877
		MaxSub	0.104	0.168	0.174			
		Procheck (%)	76.90	79.00	70.50			
8	B9WFW8	LG Score			1.564	Phyre	1SXJ	0.334
		MaxSub			0.111			
		Procheck (%)			77.20			

#### Structure analysis

Structure-based functional annotations of proteins are considered to be a more reliable approach than sequence-based function assignment, as the structures of homologous proteins are more conserved in evolution than sequences (Hassan and Ahmad 2011). We used various tools for the analysis of predicted structure of HPs. Results of Pocket-Finder (Laurie and Jackson 2005), information from literature for the selected templates and docking analyses were used to define the catalytic sites. The ProFunc server was used to identify the structural motifs associated with the biological functions (Laskowski et al. 2005). Furthermore, DALI server was used for the function prediction (Holm and Rosenstrom 2010). Docking simulation study was carried out with AutoDock 4.2 (Morris et al. 2009) using standard protocol. PASS method was used to define the active site of HPs, and the center of mass was calculated for the predicted catalytic cavity (Brady and Stouten 2000). The docking simulations end with multiple runs, and cluster analysis of ligands was performed with their corresponding docked energy. Docking solutions with ligand atom root mean square deviation (RMSD) within 2.0 Å of each other were clustered together and ranked by the lowest-energy representative (Maiorov and Crippen 1994). The lowest-energy solution of the lowest ligand all-atom RMSD cluster was accepted as the calculated binding energy. The top-posed docking conformations were obtained and post-docking energy minimization carried out with Discovery Studio 3.5. The resultant structure files were analyzed using PyMOL visualization programs (Lill and Danielson 2011).

## Result and discussion

Functional annotation of HPs is essential for better understanding of biological processes at systems level and predicting the behavior of biological system for designing a predictive disease model (Mazandu and Mulder 2012). For this, various novel methods such as neural network model, support vector machine and hidden Markov model-based tools have been developed recently (Rashid et al. 2007). These methods are efficient, intelligent and can complement the classical homology search, to detect functional constraints on genome evolution. However, the characterization of protein function in the context of their structure is more intuitive, reliable and generally more applicable to extract the biochemical or enzymatic function (Haas et al. 2011). Here, we annotated the functions of HPs of *C. dubliniensis* based on sequence and structure analysis (Table 3). We found that these HPs possess transport, kinase, transferase, ketosteroid, isomerase, hydrolase, oxidoreductase activity and DNA- and RNA-binding properties. A detailed analysis of each structure is presented here separately.

**Table 3:** Function of HPs of *C. dubliniensis*

S. no.	Gene ID	Uniprot ID	Protein product	Protein function
1	8047379	B9WFH2	XP_002419776.1	Transporter activity
2	8047381	B9WFH4	XP_002419778.1	Kinase activity
3	8047468	B9WFR9	XP_002419873.1	Transferase activity
4	8047469	B9WFS0	XP_002419874.1	Ketosteroid isomerase activity
5	8047470	B9WFS1	XP_002419875.1	Hydrolase activity
6	8047474	B9WFS6	XP_002419880.1	RNA binding
7	8047491	B9WFU3	XP_002419897.1	Oxidoreductase activity
8	8047669	B9WFW8	XP_002419922.1	DNA binding

### HP B9WFH2

Sequence-based analysis suggests that HP B9WFH2 may act as a transporter protein (Table S1). Conserved domain analysis strongly suggests that this protein belongs to the major facilitator superfamily (MFS), has multi-domain for sugar transporter-like protein and may be involved in transport activity (Marchler-Bauer et al. 2011) (Table S2). HHpred also suggests high similarity with α-helical transmembrane protein glucose transporter (Soding et al. 2005) (Table S3). Motif search using InterProScan suggests that HP B9WFH2 sequence possesses a motif involved in sugar/inositol transporter (Quevillon et al. 2005). Based on these observations, we suggest that HP B9WFH2 may function as a transporter protein.

**Fig. 1:**
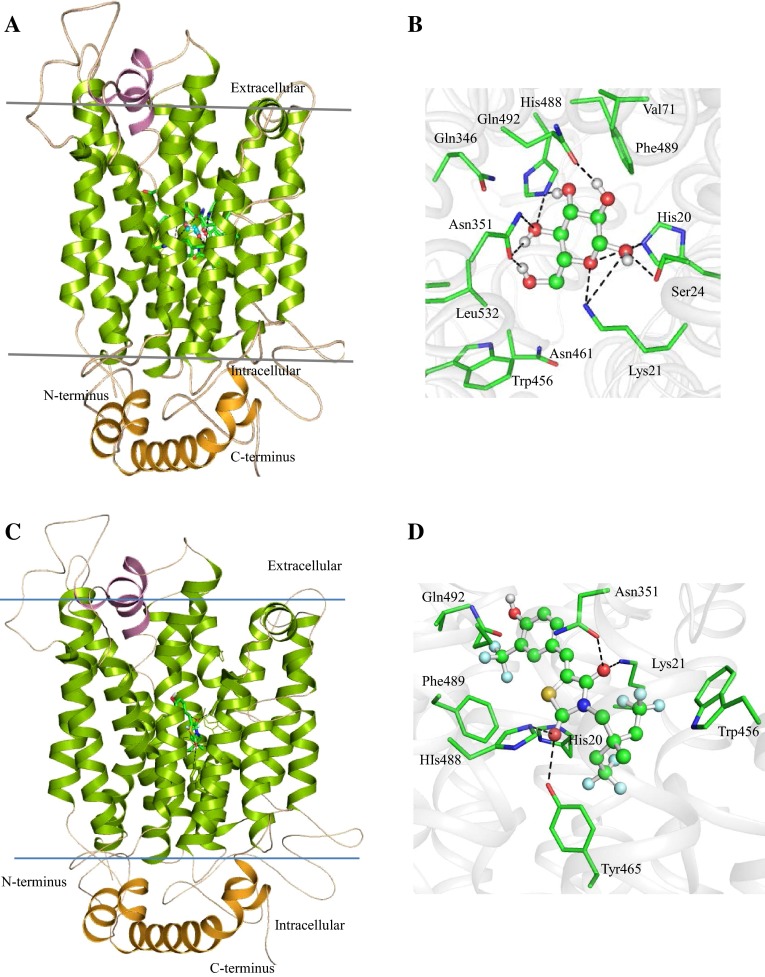
The structure of HP B9WFH2 bounded to BGC. **a** The overall structure of HP B9WFH2 comprised 15 transmembrane helices (*green*), 2 small extracellular helices (*pink*) and 4 intracellular helices interconnected (*orange*) with loops. **b** BGC docked in the HP B9WFH2. The active site residues are shown in stick and BGC in ball and stick model. The hydroxyl groups of BGC are involved in polar interaction (*black dotted lines*) with residues Lys21 and His20, Aromatic residues Phe489 and Trp456 present in the vicinity of BGC may involve in regulation glucose transport. **c** Showing GLUT inhibitor (5-(4-hydroxy-3-trifluoromethyl benzylidene)-3-[4,4,4-trifluoro-2-methyl-2-(2,2,2-trifluoroethyl) butyl] thiazolidine-2,4-dione) binds HP B9WFH2 at the same region where BGC binds to the HP B9WFH2. **d** Residues interacts with the GLUT inhibitors are shown in stick model

We predicted the three-dimensional structure of HP B9WFH2 using tool MODELLER, SWISS-MODEL and Phyre (Eswar et al. 2006; Kelley and Sternberg 2009; Kiefer et al. 2009). Analyses of these predicted structures indicate that the structure predicted by Phyre is the best among all three models with 83.60 % of the residues found in the allowed region of the Ramachandran (RC) plot (Laskowski et al. 1996) (Table 2). The RMSD of the model with respect to the template (PDB code: 4GBZ) is 2.419 Å, indicating similar functionality (Maiorov and Crippen 1994). The overall structure of HP B9WFH2 comprises 21 α-helices, of which 15 are transmembrane, 4 helices are intracellular and 2 are extracellular (Fig. 1a). Like other MFS transporter protein structure, the N- and C-terminal and seven-helix bundles come together to form a ‘V’-shaped transporter (Sun et al. 2012).

Docking result showed that residues His20, Lys21, Ser24, Asn351, His488 and Gln492 are involved in polar interaction with β-d-glucose (BGC) and hydroxyl group of BGC specifically recognized through the total seven H-bonds (Fig. 1b). The aromatic residues, Trp456 and Phe489, present in the vicinity of the BGC, are surrounded by Val71, Gln346, Asn461 and Leu532. These residues are significant for biological activity and contribute to the spatial fitting BGC (Sun et al. 2012). Hence, this motif is amenable to drug targeting. Structure alignment with the template showed that residues Gln346 and Asn351 are conserved in the model and important for interaction with BGC (Figure S1). The polar interaction between TMs and ICs residues regulated the passage of BGC. H-bond interaction results are consistent with glucose transporter protein (XylE), and residues of TMs (Glu190 and Arg477) and ICs (Ser264, Trp267, Asn580, Gln583, Tyr584, Glu585) are conserved in the protein of this family (Figure S2). It has been reported that mutation in these residues led to a substantial loss in the active uptake of D-xylose into the cell, implicating that intracellular helix domain has a critical role and contributes to proton symport (Sun et al. 2012).

Furthermore, ProFunc (Table 4) server predicted SP, MFS_gen_substrate_transporter and sugar transport-conserved motifs showing close resemblance with that of MFS protein (Laskowski et al. 2005). The other two structural motifs, Val48-Ser51 and Val152-Ile155, are also depicted to be conserved in HP B9WFH2 and may have a similar function. We identified a structurally similar protein to HP B9WFH2 on DALI server (Holm and Rosenstrom 2010). Our results clearly indicate a significant similarity with the glycerol-3-phosphate transporter (*Z* score = 56.5), d-xylose-proton symporter (*Z* score = 26.8) and many other proteins listed in Table S4. All these analyses strongly suggest that B9WFH2 may acts as a transporter protein.

**Table 4:** Sequence and structural motifs present in the HPs

S. no.	Uniprot ID	Sequence motif	Structure motif
1	B9WFH2	MFS_gen_substrate_transporter	Val48-Ser51
		Sugar transport	Val152-Ile155
2	B9WFH4	Seg	Asn146- Leu148
		DAGK_cat	Gly209- His211
		Sphingosine kinase	Gln344- Arg346
		DAGK	Thr95- Ile97
			Leu152-Ile154
			Ser167- Lys169
3	B9WFR9	CoA-transferase family III (CaiB/BaiF)	Tyr274- Ala276
		Alpha Methylacyl-Coa racemase	Gly334- Ile336
4	B9WFS0	–	–
5	B9WFS1	α/β-Hydrolase	Leu86-Leu88
6	B9WFS6	Methyltrn_RNA_3 Nucleic acid-binding proteins	Ile212-Glu214
7	B9WFU3	Thioredoxin	Asp145-Lys147
8	B9WFW8	Rad17	Gly101-Ser105
			Asn70-Thr72
			Glu193-Glu195
			Gln17-Asp179
			Thr597-Gly599

To further validate this protein as a potential drug target, we docked a novel glucose transport (GLUT) inhibitor (5-(4-hydroxy-3-trifluoromethylbenzylidene)-3-[4,4,4-trifluoro-2-methyl-2-(2,2,2-trifluoroethyl) butyl]thiazolidine-2,4-dione) in the glucose binding site of HP B9WFH2 (Fig. 1c). This compound has been reported to show antitumor potency by suppressing glucose uptake and occupied similar spatial orientation at HP B9WFH2 and provide steric hindrance to the binding of BGC (Eswar et al. 2006). It is noteworthy that the oxygen of the two keto-groups of thiazole ring of GLUT inhibitor shows polar interaction with the proposed active site residues, Lys21, Asn351, Tyr465 and His488, of HP B9WFH2 (Fig. 1d). Moreover, other residues surround the GLUT inhibitor are Trp456, Phe489 and Gln492. These observations clearly indicate a possibility of HP B9WFH2 as a potential target for antifungal therapy.

### HP B9WFH4

Analysis for sub-cellular localization of HP B9WFH4 indicates its presence in the nuclear region. Signal peptide prediction suggests that this is a non-secretory protein without any transmembrane helix (Table S1). Domain analysis results reveal that HP B9WFH4 possesses diacylglycerol (DAG) kinase catalytic domain including sphingosine kinase domain, responsible for the kinase activity (Marchler-Bauer et al. 2011) (Table S2). HHpred also suggests high similarity with DAG kinase protein (Laskowski et al. 2005) (Table S3). InterProScan results show that HP B9WFH4 sequence possesses motif involved in activation of protein kinase C activity by G-protein coupled receptor signaling pathway (Lubec et al. 2005). Based on these observations, we suggest that HP B9WFH4 may function as a kinase protein. The virulence factor analysis shows that HP B9WFH4 has an adhesin character; hence it may be involved in virulence as well (Chaudhuri et al. 2011).

**Fig. 2:**
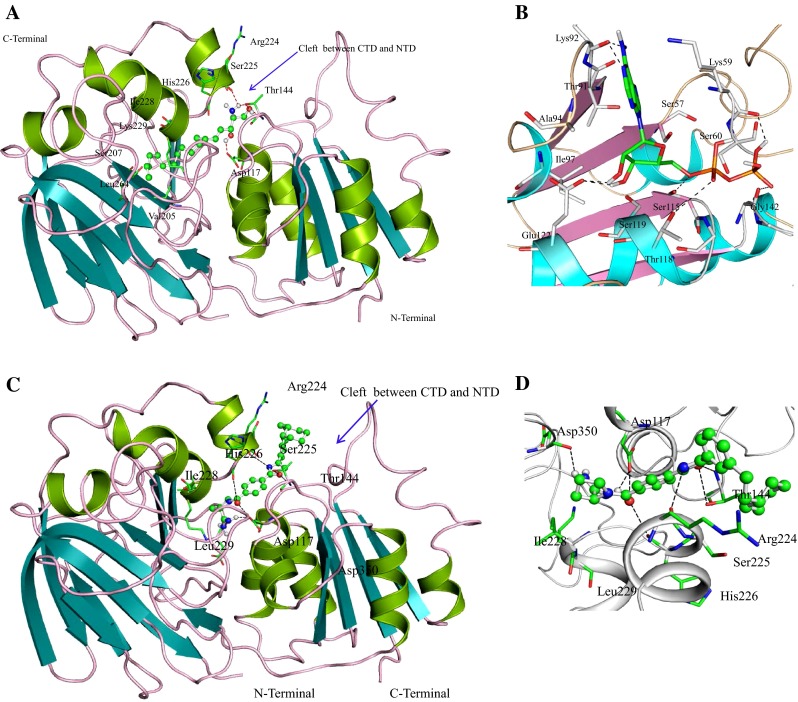
Overall structure of HP B9WFH4. **a** The structure of HP B9WFH4 adopts two domains architectures, CTD designated for sphingosine binding, whereas ADP binds in the NTD. The hydrophobic pocket of CTD comprises residues Val205, Ile228 and Leu264 which are involved in hydrophobic interaction with polycarbon tail of sphingosine. The polar head of sphingosine is spatially fitted at the cleft of CTD and NTD. Residues Asp117, Thr144 and Ser225 are involved in polar interactions (shown in *red dots*). **b** Docked pose of ADP at NTD. The important residues of HP B9WFH4 are shown in stick and H-bond interactions are denoted by *black dashed lines*. **c** Showing sphingosine kinase inhibitor [(S)-1-(4-(4-(3-(2-Cyclohexylethyl) phynyl)oxazol-2-yl)benzoyl) pyrrolidine-2carboximidamide] binds HP B9WFH4 at the same region where sphingosine binds to HP B9WFH4. **d** Residues that interact with the sphingosine kinase inhibitor are shown as stick model

We predicted the model of HP B9WFH4 on SWISS-MODEL with diacylglycerol kinase (PDB ID: 3T5P) as a template (Kiefer et al. 2009). The predicted structure was validated at SAVES server showing 83.80 % of residues are in the allowed region of the RC-plot (Table 2). The RMSD of the model with respect to template was 2.389 Å, which is quite good and indicates similar functionality (Maiorov and Crippen 1994) (Table 2). The overall structure comprises eight α-helices and 14 β-strands, resembling the dinucleotide-binding motif of the Rossmann fold (Wang et al. 2013) (Fig. 2a). Another structurally related protein to HP B9WFH4 is sphingosine kinase 1 (SphK1) (PDB ID: 3VZB), having RMSD 3.712 Å, which is a lipid kinase that catalyzes the conversion of sphingosine to sphingosine-1-phosphate (Wang et al. 2013).

These enzymes play a role in lymphocyte trafficking, angiogenesis and response to apoptotic stimuli (Wang et al. 2013). Therefore, sphingosine is used as ligand for docking study with HP B9WFH4 to explicit active site residues and their functionality. It binds at the C-terminal domain (CTD) of SphK1 which catalyzes sphingosine to sphingosine-1-phosphate. The central role of SphK1 enzyme is in modulating the S1P levels in cells; it emerged as an important regulator for diverse cellular functions and may be a potential target for drug discovery (Wang et al. 2013). SphK1 consists of a two-domain architecture, CTD and NTD (N-terminal domain), and its catalytic site is located in the cleft between these two domains, and a hydrophobic lipid-binding pocket is buried in the CTD. A similar structural topology is observed in HP B9WFH4. The spatial orientation and docking interactions of sphingosine with HP B9WFH4 are consistent with SphK1 enzyme (Fig. 2a). The hydrophilic 2-amino-1,3-diol moiety head of the sphingosine spatially fitted at the cleft between two domains, making hydrogen bond interactions with Asp117, Thr144 and Ser225. The hydrophobic pocket of CTD comprises residues Val205, Ile228 and Leu264, which are involved in hydrophobic interaction with the polycarbon tail of sphingosine. Binding of sphingosine to SphK1 is mediated by anchoring of the hydrophilic head group to the protein surface and accommodation of the hydrophobic alkyl chain in the interior of protein. Further, the active site residue Asp117 is conserved in both proteins. Structural alignment of the HP B9WFH4 with the template structure reveals that Asp117 is conserved and may play a crucial role in the function of the HP B9WFH4 (Figure S3).

To further validate the role of this protein as a drug target, we performed a docking study of HP B9WFH4 with amidine-based sphingosine kinase inhibitor [(S)-1-(4-(4-(3-(2-cyclohexylethyl) phynyl)oxazol-2-yl)benzoyl)pyrrolidine-2carboximidamide] which is a potential drug for leukemia (Kennedy et al. 2011). We found that amidine-based sphingosine kinase inhibitor binds in the same cavity where sphingosine binds (Fig. 2c). The oxygen of oxazole ring of SphK inhibitor is involved in polar interaction with Thr144 and nitrogen of the oxazole ring is involved in polar interaction with Arg224, whereas the polar head of the inhibitor is involved in polar interaction with Asp117, His226 and Asp350 (Fig. 2d). Other important residues present in the vicinity of SphK inhibitor are Ile228 and leu229. These observations clearly indicate a possibility of HP B9WFH4 as a novel therapeutic target because of a close target similarity (Kennedy et al. 2011).

We further compared the nucleotide binding in HP B9WFH4 with SphK1, and docking of ADP was carried out to validate the structure. The active site at NTD comprises five α-helices and four parallel β-sheets. ADP binding showed that adenine ring fits at the cleft consisting of Ser57-Arg64 and Thr91-Ile97, as in the SphK1, and Lys92 at the opening of the active site is involved in H-bond interaction. Both of the ribose hydroxyls are involved in polar interaction with Glu122. The side chain of ADP, α-phosphate is hydrogen bonded with Ser115 and Thr118, whereas β-phosphate forms two H-bond interactions with Gly142 and Lys59 (Fig. 2b). It has been observed that ADP binding site of HP B9WFH4 is consistent with the crystal structure. However, π-stacking interactions with the backbones of Glu55 and Arg56 and with side chain Asn22 are not observed here.

ProFunc analysis identified sphingosine kinase and DAGK motifs (Laskowski et al. 2005). Six other structural motifs were also identified as Asn146-Leu148, Gly209-His211, Gln344-Arg346, Thr95-Ile97, Leu152-Ile154 and Ser167-Lys169 (Table 4). DALI results showed a significant similarity with other kinase protein ‘BmrU protein’ with maximum *Z* score = 43.8 for the top five hits (Holm and Rosenstrom 2010) (Table S4). These results also suggest the possible kinase activity of HP B9WFH4.

### HP B9WFR9

HP B9WFR9 is predicted as a cytoplasmic protein. Domain analysis suggests the similarity with CoA-transferase family III that has multi-domain for acyl-CoA-transferases and carnitine dehydratase (Marchler-Bauer et al. 2011) (Table S2). Results of HHpred showed higher homology with FCOCT (PDB ID: 3UBM), a bacterial formyl-CoA:oxalate CoA-transferase protein (Soding et al. 2005) (Table S3). InterProScan result also suggests that the HP B9WFR9 sequence possesses a motif that is involved in formyl-CoA transferase activity (Quevillon et al. 2005).

**Fig. 3:**
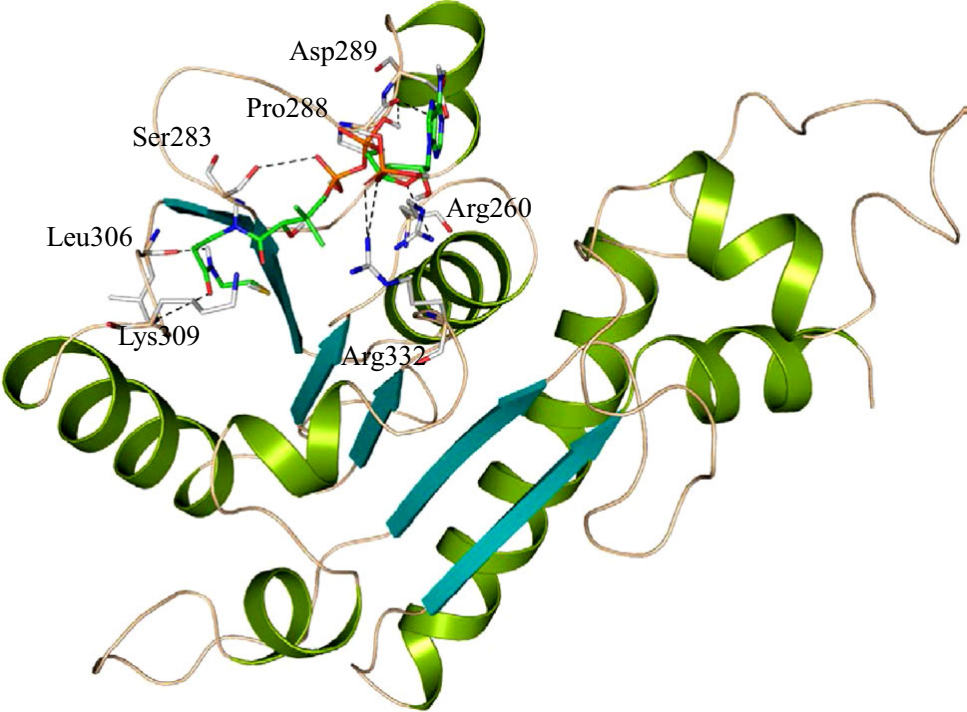
Overall structure of HP B9WFR9. Structure of B9WFR9 docked with coenzyme A (CoA). Predicted active site residues are shown in stick and polar interaction are represented with *dashed line* (*black*)

We successfully predicted the structure of HP B9WFR9. The best model having 87.30 % of residues in the allowed region of the RC-plot (Table 2) is selected for further analysis. The structure of HP B9WFR9 shows a close resemblance with the template, formyl-coenzyme A (CoA):oxalate CoA-transferase, from the acidophile *Acetobacter aceti* with an RMSD of 0.178 Å (Mullins et al. 2012). The overall structure of HP B9WFR9 comprises eight α-helices and six parallel β-sheets having very similar backbone topology with the template (Fig. 3). In the template Rossmann fold domain with a conserved small domain contributes to the formation of active site for the catabolic disposal of carboxylic acid (Mullins et al. 2012).

To further validate the functional features, we performed docking study with template ligand CoA. Molecular docking results showed that the binding site of HP B9WFR9 consists of majorly α-helices (α3, α4 and α6) and extended from α3 to α6 to form a cleft-like structure, and the surrounding loops are rich in positively and negatively charged residues (Fig. 3). The predicted active site residue Arg260, Ser283, Pro288, Asp289, Leu306, Lys 309 and Arg332 are found to be involved in H-bonding. Among active site residues, Arg332 and Lys309 are structurally conserved and essential for electrostatic interactions (Mullins et al. 2012) (Fig. 3 and Figure S4).

Functional motif search resulted in the identification of two biologically significant motifs in the HP B9WFR9 (Table S5), CoA-transferase family III (CaiB/BaiF) and α-methylacyl-Coa racemase with two structurally significant motifs, Tyr274-Ala276 and Gly334-Ile336. DALI search result is consistent with ProFunc finding and showed a significant similarity with the formyl-coenzyme-A transferase (*Z* score = 47.1) (Table S4). These findings clearly indicate the possible transferase activity of B9WFR9 (Holm and Rosenstrom 2010; Laskowski et al. 2005).

### HP B9WFS0

**Fig. 4:**
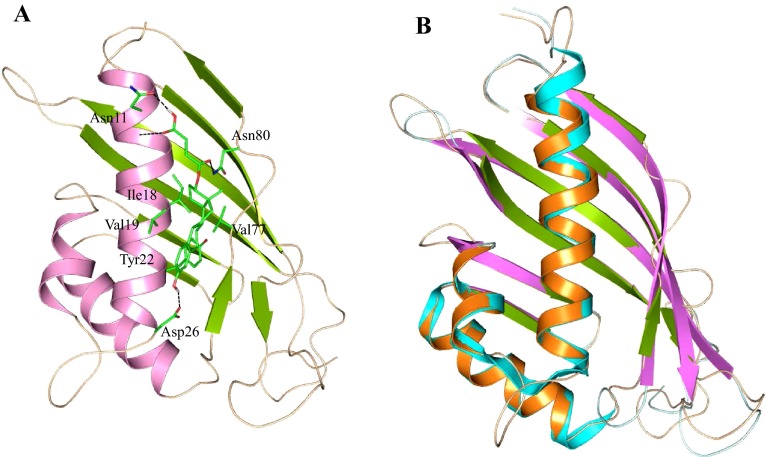
**a** Overall structure of HP B9WFS0 docked with estradiol-17 β-hemisuccinate. **b** Superimposed structure of HP B9WFS0 with its template (PDB id: 2RGQ)

HP B9WFS0 was predicted as a cytoplasmic protein and possibly possesses ketosteroid isomerase activity (Table S1 and Table S3). To explore its function, a model of B9WFS0 was generated using MODELLER (Eswar et al. 2006). Procheck results showed that 85.80 % of residues are in the allowed region of the RC-plot and LG and MaxSub score are 2.341 and 0.309, respectively (Laskowski et al. 1996; Wallner and Elofsson 2003). The predicted structure was superimposed with the template (PDB ID: 2RGQ) and showed an RMSD of 0.46 Å (Table 2). The overall structure of HP B9WFS0 comprised three α-helices and eight parallel and anti-parallel β-strands, similar to the crystal structure of Rv0760c protein from *M. tuberculosis* (Cherney et al. 2008). The active site of HP B9WFS0 is very similar to a related protein of *M. tuberculosis* and resembles a cone-shaped closed-barrel fold formed by a curved anti-parallel β-sheet composed of seven β-strands and four adjacent anti-parallel α-helices (Fig. 4).

HP B9WFS0 is primarily involved in steroid metabolism and found to be an important target for antibiotic development. The active site of this enzyme lies at the surface, just above the longest helix and has clearly two distinct hydrophobic and hydrophilic binding domains (Fig. 4). The ligand estradiol-17 β-hemisuccinate spatially fitted at the surface and its hydrophilic tail showed polar interaction with Asn11 and Asn80, whereas the hydrophobic head protruded deep to the lower side of the pocket and showed π–π interaction with Tyr22. Other residues Val19, Val77 and Ile18 are involved in hydrophobic interaction. However, the hydroxyl group of benzene at the head of estradiol-17β-hemisuccinate showed polar interaction with Asp26 to form a tri-point attachment with protein. Other protein function analysis showed a significant similarity with the γ-hexachlorocyclohexane dehydrochlorinase and ketosteroid isomerase protein (Eberhardt et al. 2013).

### HP B9WFS1

**Fig. 5:**
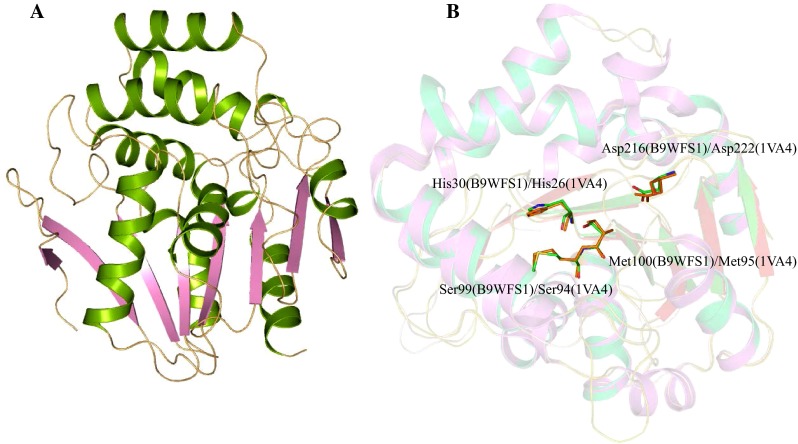
**a** Overall structure of HP B9WFS1. **b** Superimposed structure of HP B9WFS1 with template structure showing conserved residues

HP B9WFS1 is found to be present in the cytoplasm (Table S1) and is involved in the lysophospholipase activity. This protein belongs to the α/β-hydrolase family and may have hydrolase activity (Table S2). This result is consistent with HHpred and InterProScan results described in Table S3. The structure of HP B9WFS1 was predicted with MODELLER. The refined structure shows 86.60 % of the residues in the allowed region of the RC-plot with LG and MaxSub score of 5.560 and 0.533, respectively. The structure of HP B9WFS1 showed a profound similarity with the aryl esterase from *Pseudomonas fluorescence* (Cheeseman et al. 2004) with RMSD of 0.296 Å (Fig. 5a). The structure of this model totally resembles along with the catalytic triad, His30, Ser99, Met100 and Asp216 (Fig. 5b). Ser97 is also located in the canonical elbow of the conserved sequence Gly-X_1_-Ser-X_2_-Gly, similar to esterases and lipases family (Cheeseman et al. 2004). In our model, X_1_ is His98 and X_2_ is Met100, the same as the template protein; however, Gly96 is substituted by Ser101. The structure alignment of HP B9WFS1 with its template protein depicts that in the acyl-binding pocket, only Val125 is conserved and no other residue is conserved in the alcohol-binding pocket (Fig. 5b).

This type of lipase has been reported in soil bacteria showing activity in response to oxidative stress. Esterase and lipase efficiently act in ester hydrolysis, while the generation of peroxycarboxylic acid is efficiently performed by haloperoxidases. The model closely resembles the *Pseudomonas fluorescens* aryl esterase structure, which is functionally more similar to the esterases and haloperoxidases. Therefore, we can say that HP B9WFS1 may function as an esterase protein. Further, functional analysis using ProFunc and InterPro database scan revealed the presence of α/β-hydrolase motifs in the HP B9WFS1 (Table 4) with structural motifs Leu86-Leu88. DALI results also showed similarity with arylesterase with *Z* score of 49.4. All these systematic analyses precisely suggest that HP B9WFS1 may have hydrolase-like activity and be essential for survival of the pathogen.

#### HP B9WFS6

HP B9WFS6 was found to be distributed in the nuclear region and might be involved in methyltransferase activity (Table S1). There domains are highly conserved with RNA methyltransferase family (Table S2). Similar results are obtained with HHpred analysis, and these show high similarity with rRNA methylase, a methyltransferase (Table S3). HP B9WFS6 also acts in nucleotide binding. These findings are crucial and suggest the role of HP B9WFS6 in methyltransferase and nucleic acid-binding activity (Marchler-Bauer et al. 2011; Quevillon et al. 2005).

**Fig. 6:**
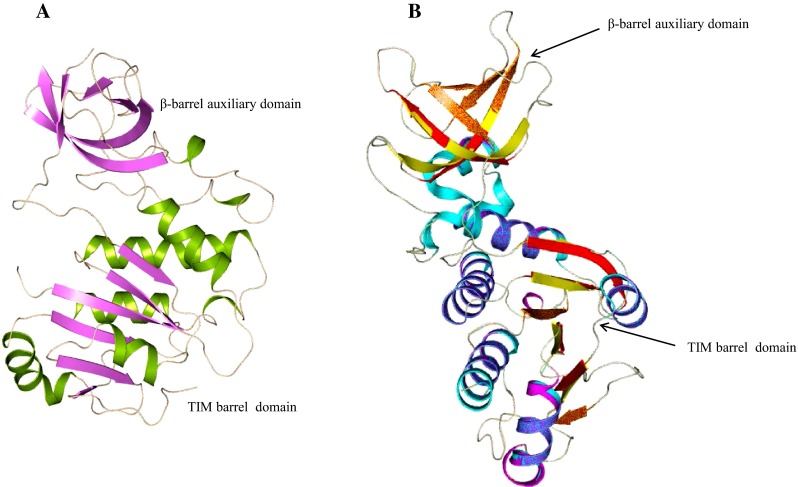
**a** Overall structure of B9WFS6 showing TIM barrel domain and β-barrel auxiliary domain. **b** Superimposed structure of HP B9WFS6 with template structure

Furthermore, we predicted the three-dimensional structure of HP B9WFS6 to explain its function more precisely. The stereochemical quality of refined model show 85.10 % of the residues in the allowed region of the RC-plot (Table 2), with LG score 5.205 and MaxSub score: 0.367. This protein shows structural similarity with *MT1* protein from *M. thermoautotrophicum* (Zarembinski et al. 2003). The overall structure of HP B9WFS6 comprises eight α-helices and seven β-sheets (Fig. 6a). *MT1* protein consists of two subunits, the small β-barrel auxiliary domain (MT1-CSD) and larger domain defined as the TIM barrel (MT1-DD). The TIM barrel is essential for interfacial contact to form the homodimer of the *MT1* protein. The overall structure of MT1-DD resembles the classical nucleotide-binding Rossmann fold and is highly conserved in HP B9WFS6. The small domain of HP B9WFS6 shows a similar topology to MT1-CSD; however, their sequences are not highly conserved. These two domains are almost conserved in most of the bacteria, yeast and archaebacteria. Interestingly, the TIM barrel domain structure is generally conserved in various organisms, although their sequences are highly divergent and give rise to a plethora of distinct functions (Zarembinski et al. 2003). Structural alignment showed the presence of nucleotide-binding Rossmann fold in HP B9WS6; however, sequences were not conserved. HP B9WS6 has conserved residues Asp230 and Asn351 near the site of knot formation, which is distinct to the MT1 protein (Fig. 6b). The predicted structure of B9WFS6 also contains a β-barrel auxiliary domain, which is structurally similar to CspA protein of *E. coli,* an RNA chaperone that binds to RNA to prevent hairpin formation for transcription anti-termination. ProFunc server revealed the presence of methyltrn_RNA_3 and nucleic acid-binding motifs in the HP B9WFS6 with one structural motif (Ile212-Glu214) (Table 4). DALI result shows similarity with the methyl transferase from the *M. thermoautotrophicum* (*Z* score = 41.0), and *E. coli* (*Z* score = 13.5) (Table S4). These findings are quite helpful to assign RNA-binding activity to HP B9WFS6.

#### HP B9WFU3

Domains of HP B9WFU3 are conserved with the protein disulfide isomerase (PDIa) family. It also contains s multi-domain that shows similarity with protein disulfide oxidoreductases and thioredoxin fold, involved in isomerase activity (Table S2). Similar prediction is obtained with Hpred analysis and shows high similarity with disulfide isomerase and thioredoxin-like fold (Table S3). InterProScan result also suggests the presence of thioredoxin-like fold in HP B9WFU3 which maintains cell redox homeostasis.

**Fig. 7:**
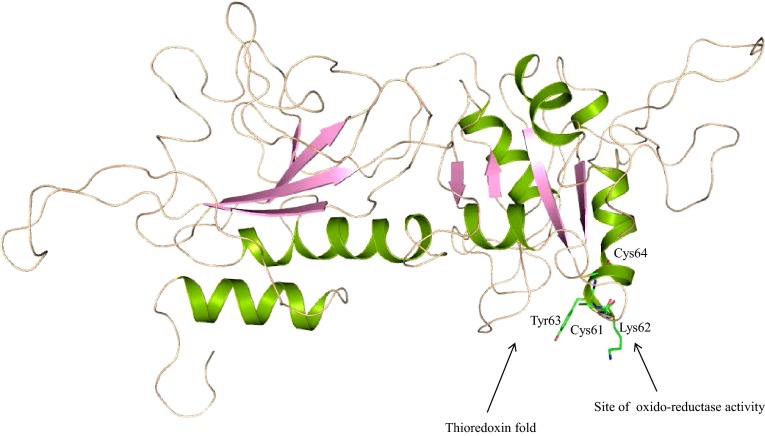
Overall structure of HP B9WFU3 having two units of thioredoxin fold; the N-terminal unit having conserved residues may be the site of oxidoreductase activity

The three-dimensional structure of HP B9WFU3 was predicted to understand their function at the molecular level. The refined model shows 76.90 % of the residues in the allowed region of the RC-plot. The ProQ module results are impressive with an LG score of 1.123 and MaxSub score of 0.104, which are considerable scores to carry out further analysis. The overall structure of HP B9WFU3 comprises four α-helices (Fig. 7) and shows a close RMSD 1.877 Å with the template protein, disulfide isomerase ERp72 (Kozlov et al. 2010). This protein belongs to the PDIa family and is responsible for catalyzing the proper oxidation and isomerization of disulfide bonds of newly synthesized proteins in the endoplasmic reticulum. The overall structure of the PDI family proteins possesses a characteristic “U” shape twisted structure formed with four thioredoxin folds. However, our model contains only two thioredoxin folds. The prototypical thioredoxin fold is composed of a five-central β-sheet sandwiched with two α-helices on each of the other sides, whereas the predicted structure has only four β-strands. The active site of the template of HP B9WFU3 is characterized by the C-G-H-C consensus sequences located at the N-terminus of the second α-helix, consistent with PDI family proteins. However, in the predicted structure, GlyL92 and His93 have been mutated by Lys62 and Tyr63. These two cysteines are responsible for oxidoreductase activity (Figure S5). In the oxidized state, the two cysteines form an intramolecular disulfide bond that in principle enables PDI to convert a pair of sulfhydryl groups of the polypeptide substrate into a disulfide bond. Similarly, PDI-like protein ERp57, reported in ER of *Saccharomyces cerevisiae,* interacts with two lectin chaperons, calnexin and calreticulin, and promotes the oxidative folding of newly synthesized glycoprotein. Other prediction tools also supported the presence of thioredoxin domain in our model (Table 4 and Table S4), suggesting the possible thioredoxin function of HP B9WFU3.

#### HP B9WFW8

HP B9WFW8 is localized in the nuclear region of the cell (Table S1). CDD result suggests that this protein has a conserved multi-domain similar to cell cycle checkpoint protein Rad17 involved in DNA damage control (Table S3) (Nakai and Horton 1999). HHpred predicted the processivity clamp, DNA sliding clamp and AAA+ polymerase fold in HP B9WFW8 reported to be involved in DNA replication, recombination and restriction (Table S3) (Soding et al. 2005). InterProScan result shows the presence of similar motifs belonging to cell cycle regulatory proteins of yeast. These proteins are crucial in cell cycle regulation and control the DNA damage (Quevillon et al. 2005). Therefore, the structure of HP B9WFW8 is predicted to define their probable functions more precisely.

**Fig. 8:**
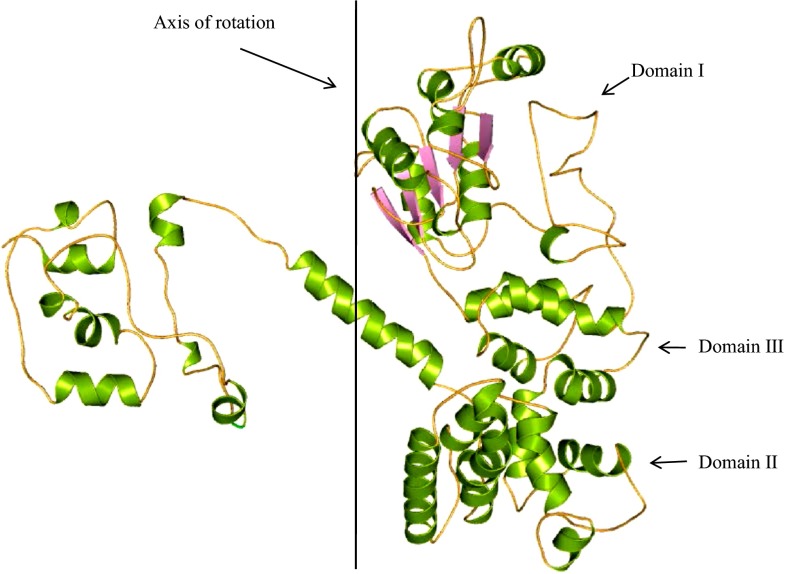
Overall structure of HP B9WFU3. *Line* shown in the figure is the line across which DNA winds, and three domains (label *I*, *II* and *III*) form complex for DNA binding, having similar topology to the RFC monomer unit

The overall topology of HP B9WFW8 predicted is similar to the putative methyltransferase protein of *S. cerevisiae* (Bowman et al. 2004), with an RMSD of 0.334 Å, suggesting a similar function. The overall structure of B9WFW8 comprises 28 α-helices and 5 β-strands (Fig. 8) and has clamp loader complex (replication factor-C, RFC) that binds to the sliding clamp (proliferating cell nuclear antigen, PCNA) (Fig. 8). Sliding clamps are ring-shaped proteins that encircle DNA and confer high processivity on DNA polymerases (Bowman et al. 2004). Tight interfacial coordination of the ATP analog, ATP-γS by RFC, results in a spiral arrangement of the ATPase domains of the clamp loader above the PCNA ring (Figure S6). Placement of a model for primed DNA within the central hole of PCNA reveals a striking correspondence between the RFC spiral and the grooves of the DNA double helix. Further, Profunc result revealed the presence of single motif Rad17 in HP B9WFW8 (Table 4). However, five structural motifs are also present in the structure, These are Gly101–Ser105, Asn70–Thr72, Glu193–Glu195, Gln17–Asp179 and Thr597–Gly599 probably involved in anion and cation binding site formation. DALI server was used to find the homologous structure and in identification of the RFC subunit (*Z* score = 17.4) and DNA Polymerase Accessory Protein (*Z* score = 16.9) in HP B9WFW8 (Table S4). These results are decisive for structure-based function determination and suggest the role of HP B9WFW8 in DNA replication.

## Conclusions

Function prediction of the putative uncharacterized protein from sequence generated by genome project is a major challenging task. Here, we have used in silico techniques to examine *C. dubliniensis* genome and exemplify the functions for HPs. Our primary sequence-based analysis led to the identification of eight HPs as biologically significant, which might be involved as enzymes (kinase, hydrolase, transferase, isomerase and oxido-reductase), transporter protein and glucose symporter and involved in cell cycle control mechanisms. Furthermore, we successfully predicted the structure of all eight HPs to describe their functions at the molecular level. The outcome of the present study may facilitate better understanding of the mechanism of virulence, drug resistance, pathogenesis, adaptability to host, tolerance for host immune response and drug discovery for treatment of *C. dubliniensis* infections.

## Electronic supplementary material

Below is the link to the electronic supplementary material.

Supplementary material 1 (DOCX 2044 kb)

## Abbreviations

HP: Hypothetical protein
BLAST: Basic local alignment search tool
PSI-BLAST: Position-specific iterative basic local alignment search tool
CDD: Conserved domain database
SMART: Simple modular architecture research tool
PANTHER: Protein analysis through evolutionary relationships
SVM: Support vector machine
PP2C: Protein phosphatase 2C
MFS: Major facilitator superfamily
CATH: Protein structure classification database
