# Supplementary material for: Structure-based functional annotation of hypothetical proteins from Candida dubliniensis: a quest for potential drug targets
Source: 3 Biotech. 2014 Oct 17;5(4):561–76. doi: 10.1007/s13205-014-0256-3 (PMC4522726; doi:10.1007/s13205-014-0256-3)
Supplement: Supplementary file 1 — Supplementary material 1 (DOCX 2044 kb) [file 13205_2014_256_MOESM1_ESM.docx]

**Structure based Functional Annotation of Hypothetical Proteins from *Candida dubliniensis:* Search for Potential Drug Target**

***Supplementary Information***

## Table S1: Subcellular localization of the protein

| S. No. | Uniprot ID | HMMTOP | SOCUI | TMHMM | SignalP | Psort |
| --- | --- | --- | --- | --- | --- | --- |
| 1. | B9WFH2 | 10 TMH | M, 10 TMH | 9 TMH | NSP | Membrane |
| 2. | B9WFH4 | NIL | S | NIL | NSP | Nuclear |
| 3. | B9WFR9 | 2TMH | S | NIL | NSP | Cytoplasmic |
| 4. | B9WFS0 | NIL | S | NIL | NSP | Cytoplasmic |
| 5. | B9WFS1 | NIL | S | NIL | NSP | Cytoplasmic |
| 6. | B9WFS6 | NIL | S | NIL | NSP | Nuclear |
| 7. | B9WFU3 | 1 TMH | M, 1 TMH | NIL | SP | Nuclear |
| 8. | B9WFW8 | NIL | S | NIL | NSP | Nuclear |

TMH*- Transmembrane Helix

# Table S2: Conserved Domain in HPs

| S. No. | Uniprot ID | Conserved Domain(super family) |
| --- | --- | --- |
| 01. | B9WFH2 | 1.Major Facilitator Superfamily (MFS),  2.Sugar (and other) transporter* |
| 02. | B9WFH4 | 1.Diacylglycerol kinase catalytic domain (DAG),  2.LCB5;Sphingosine kinase and enzymes* |
| 03. | B9WFR9 | 1.CoA-transferase family III  2.Predicted acyl-CoA transferases/carnitine dehydratase* |
| 04. | B9WFS0 | - |
| 05. | B9WFS1 | 1.Putative lysophospholipase  2.Alpha/beta hydrolase family* |
| 06. | B9WFS6 | Putative RNA methyltransferas |
| 07. | B9WFU3 | 1. Protein Disulfide Isomerase (PDIa) family  2 .Protein Disulfide Oxidoreductases and Other Proteins with a Thioredoxin fold |
| 08. | B9WFW8 | Rad17 cell cycle checkpoint protein* |

*multi-domain

**Table S3: Function Prediction on the basis of HHPred Result**

| S.No. | Uniprot ID | Similar Protein | Function | E Value |
| --- | --- | --- | --- | --- |
| 1 | B9WFH2 | 4GC0 | D-xylose-proton symport | 8E-50 |
| 2 | B9WFH4 | 3S40 | Diacylglycerol kinase | 9.8E-57 |
| 3 | B9WFR9 | 3UBM | COA:transfarase | 2E-68 |
| 4 | B9WFS0 | 2RJQ | unknown function with A cystatin-like F | 9.3e-15 |
| 5 | B9WFS1 | 3IA2 | Arylesterase; alpha-beta hydrolase fold | 5e-37 |
| 6 | B9WFS6 | 1K3R | beta barrel | 1.9e-82 |
| 7 | B9WFU3 | 3IDV | Protein disulfide-isomerase | 5e-34 |
| 8 | B9WFW8 | 1SXJ | clamp loader | 4.9e-42 |

**Table S4:** DALI result: Similar structure of HPs from *C. dubliniensis*

| S. No. | Uniprot ID | Similar structure | Z Score | Function |
| --- | --- | --- | --- | --- |
| 01. | B9WFH2 | 4GC0 | 56.5 | D-Xylose-Proton Symporter |
|  |  | 4GBY | 26.8 | D-Xylose-Proton Symporter |
| 02. | B9WFH4 | 3T5P | 43.8 | BmrU Protein |
|  |  | 3S40 | 39.7 | Diacylglycerol Kinase |
| 03. | B9WFR9 | 2VJO | 26.9 | Formyl-Coenzyme A Transferase |
|  |  | 1T4C | 47.1 | Formyl-Coenzyme A Transferase |
| 04. | B9WFS0 | 3A76 | 17.1 | Gamma-Hexachlorocyclohexane Dehydrochlorinase |
|  |  | 3D9R | 15.8 | Ketosteroid Isomerase-Like Protein |
| 05. | B9WFS1 | 3T4U | 44.8 | Arylesterase |
|  |  | 3HEA | 44.8 | Arylesterase |
| 06. | B9WFS6 | 1K3R | 41.0 | Methyl Transferase |
|  |  | 2CX8 | 13.5 | Methyl Transferase |
| 07. | B9WFU3 | 2DJ1 | 15.7 | Protein Disulfide-Isomerase |
|  |  | 4E11 | 14.6 | Protein Disulfide-Isomerase |
| 08. | B9WFW8 | 1IQP | 17.4 | Replication Factor C Subunit |
|  |  | 3U61 | 16.9 | DNA Polymerase Accessory Protein |

***
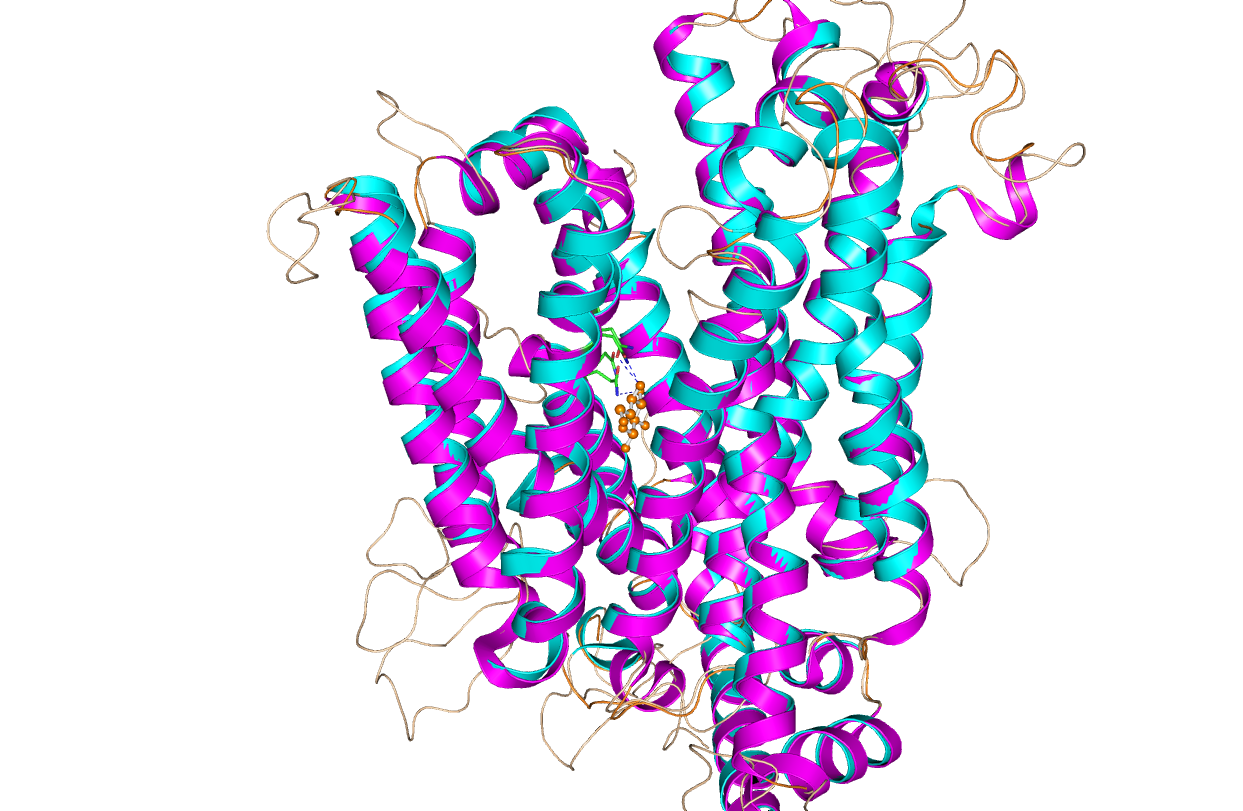
***

***Supplementary Figure S1.*** Superimposed structure of B9WFH2 and template (4GBZ). Active site residues Asn294 and Gln289 of template were found to be conserved in HP B9WFH2 with residues Asn315 and Gln346. Both are involved in polar interaction with hydroxyl group of BGC and crucial for ligand interaction in MFS protein.

***Supplementary Figure S2.*** The residues that mediate the polar interaction between the intracellular helices and TMs of HP B9WFH2 are shown in stick. Red dotted lines represent the polar interactions between the residues of ICs and TMs helices.

***Supplementary Figure S3.*** Superimposed cartoon view of HP B9WFH4 and diacylglycerol kinase (PDB ID: 3T5P. Active site residue Asp-71 (3T5P.) is conserve with Asp117 (HP B9WFH4), and proposed to act as a general base catalyst to deprotonates the sphingosine and activate the phosphoryl transfer.

***Supplementary Figure S4.*** Superimposed cartoon view of B9WFR9 with CoA-transferase protein (PDB ID: 3UBM). Here, Lys309 and Arg332 are structurally conserved with selected template active site residues (Lys81, Arg104) and are involve in polar interaction with CoA.


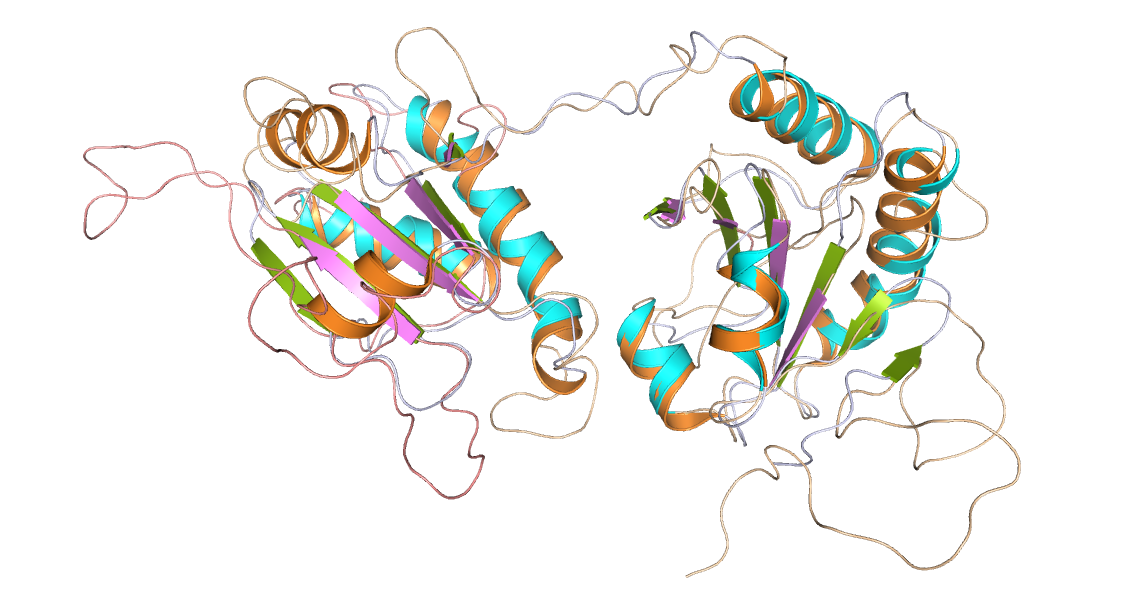


**Supplementary Fig S5.** Superimposed structure of HPB9WFS6 and its template PDB ID: 1K3R


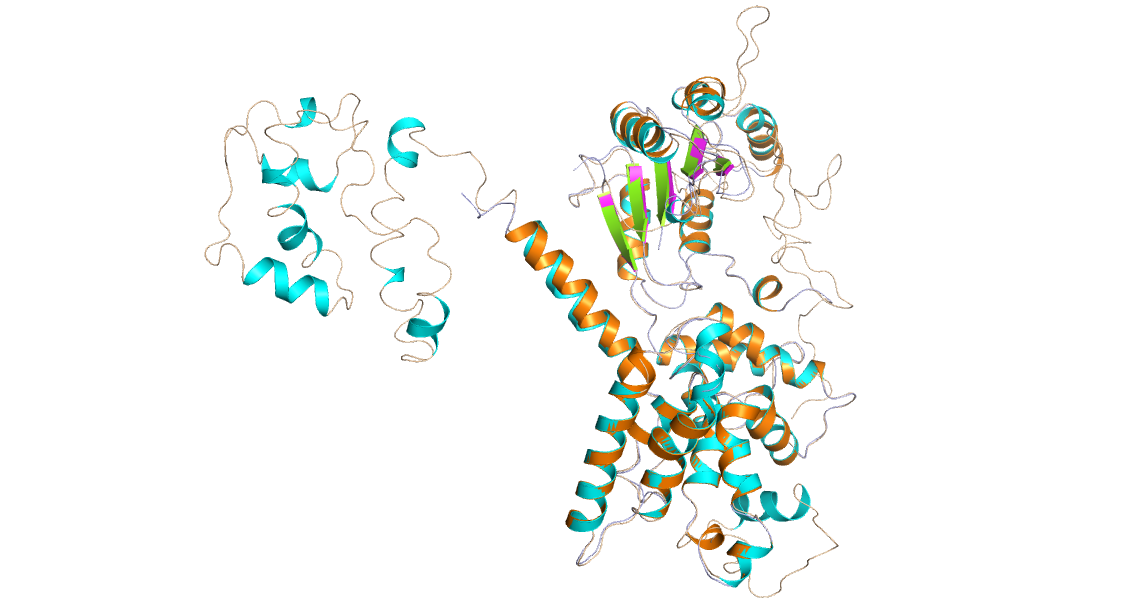


**Supplementary Figure S6.** Superimposed structure of HPB9WFS6 and its template 1K3R .
